# (±)-*N*-[4-Acetyl-5-methyl-5-(4-methyl­cyclo­hex-3-en­yl)-4,5-dihydro-1,3,4-thia­diazol-2-yl]acetamide

**DOI:** 10.1107/S1600536808004728

**Published:** 2008-02-20

**Authors:** Tebbaa Mohammed, Noureddine Mazoir, Jean-Claude Daran, Moha Berraho, Ahmed Benharref

**Affiliations:** a Laboratoire de Chimie Biomoléculaire, Substances Naturelles et Réactivité, Faculté des Sciences, Semlalia, Université Cadi Ayyad, BP 2390 Marrakech, Morocco; b Laboratoire de Chimie de Coordination, 205 route de Narbonne, 31077 Toulouse Cedex 04, France

## Abstract

The new title thiadiazole compound, C_14_H_21_N_3_O_2_S, was semi-synthesized starting from 1-(4-methyl­cyclo­hex-3-en­yl)ethanone, a natural product isolated from *Cedrus atlantica* essential oil. The stereochemistry has been confirmed by single-crystal X-ray diffraction. The thia­diazo­line ring is roughly planar, although it may be regarded as having a half-chair conformation. The cyclo­hexenyl ring has a half-chair conformation. The most inter­esting feature is the formation of a pseudo-ring formed by four mol­ecules associated through N—H⋯O hydrogen bonds around a fourfold inversion axis, forming an *R*_4_^4^(28) motif.

## Related literature

For related literature, see: Aly *et al.* (2007 ▶); Beatriz *et al.* (2002 ▶); Bernstein *et al.* (1995 ▶); Cremer & Pople (1975 ▶); Demirbas *et al.* (2005 ▶); Etter *et al.* (1990 ▶); Farghaly *et al.* (2006 ▶); Invidiata *et al.* (1996 ▶); Kubota *et al.* (1982 ▶); Nizamuddin *et al.* (1999 ▶); Ourhriss *et al.* (2005 ▶); Paolo *et al.* (2005 ▶); Radul *et al.* (2005 ▶); Sun *et al.* (1999 ▶); Udupi *et al.* (2000 ▶). 
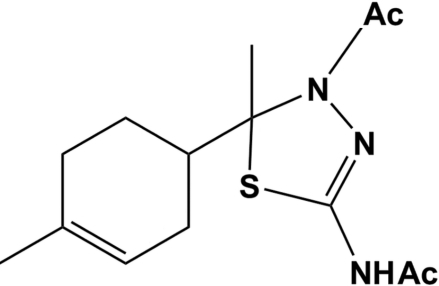


## Experimental

### 

#### Crystal data

C_14_H_21_N_3_O_2_S
*M* *_r_* = 295.40
Tetragonal, 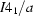

*a* = 16.6855 (3) Å
*c* = 21.8961 (8) Å
*V* = 6096.0 (3) Å^3^
*Z* = 16
Mo *K*α radiation
μ = 0.22 mm^−1^
*T* = 180 (2) K
0.29 × 0.24 × 0.08 mm

#### Data collection

Bruker APEXII CCD area-detector diffractometer
Absorption correction: none
87517 measured reflections
4637 independent reflections
3849 reflections with *I* > 2σ(*I*)
*R*_int_ = 0.032

#### Refinement

*R*[*F* ^2^ > 2σ(*F* ^2^)] = 0.037
*wR*(*F* ^2^) = 0.113
*S* = 1.11
4637 reflections
185 parameters
H-atom parameters constrained
Δρ_max_ = 0.39 e Å^−3^
Δρ_min_ = −0.26 e Å^−3^

### 

Data collection: *APEX2* (Bruker, 2006 ▶); cell refinement: *APEX2*; data reduction: *APEX2*; program(s) used to solve structure: *SIR97* (Altomare *et al.*, 1999 ▶); program(s) used to refine structure: *SHELXL97* (Sheldrick, 2008 ▶); molecular graphics: *ORTEPIII* (Burnett & Johnson, 1996 ▶), *ORTEP-3 for Windows* (Farrugia, 1997 ▶) and *CAMERON* (Watkin *et al.*, 1993 ▶); software used to prepare material for publication: *WinGX* (Farrugia, 1999 ▶).

## Supplementary Material

Crystal structure: contains datablocks I, global. DOI: 10.1107/S1600536808004728/bg2164sup1.cif

Structure factors: contains datablocks I. DOI: 10.1107/S1600536808004728/bg2164Isup2.hkl

Additional supplementary materials:  crystallographic information; 3D view; checkCIF report

## Figures and Tables

**Table 1:** Hydrogen-bond geometry (Å, °)

*D*—H⋯*A*	*D*—H	H⋯*A*	*D*⋯*A*	*D*—H⋯*A*
N3—H3⋯O1^i^	0.88	1.95	2.8223 (14)	171

Symmetry code: (i) 
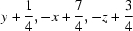
.

**Fig. 1.**
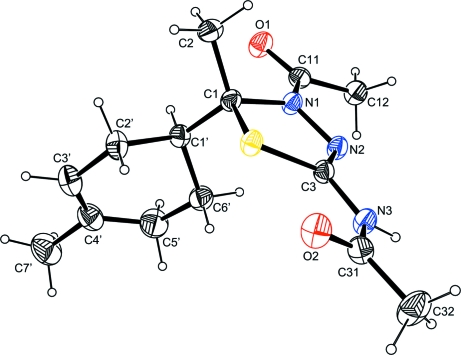
The molecular view of compound (II), showing the atom-labelling scheme. Ellipsoids are drawn at the 50% probability level.

**Fig. 2.**
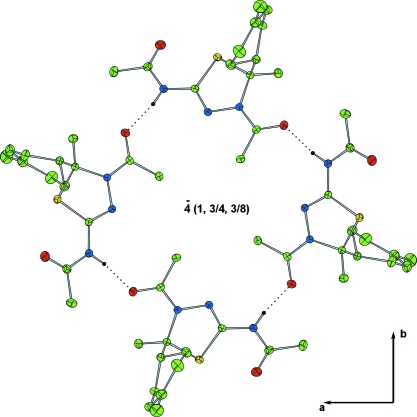
Projection down the c axis, showing the formation of the R44(28) motif through N—H···O hydrogen bonds around the fourfold screw axis 4. H atoms not involved in hydrogen bonding have been omitted for clarity.

**Fig. 3.**
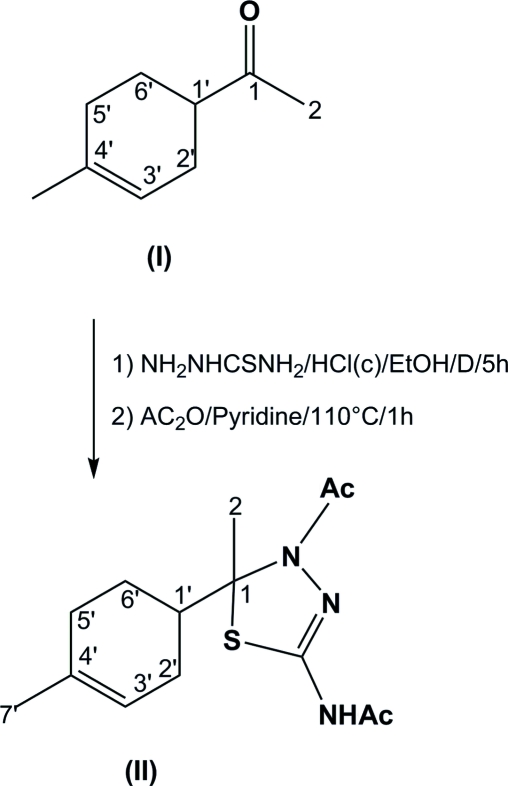
The formation of the title compound.

## supplementary crystallographic information

### Comment

Thiadiazolic compounds have beenreported in a large number of papers (Beatriz *et al.*, 2002, Farghaly *et al.*, 2006). These compounds are associated with diverse biological activities. Likewise, the 1,3,4-thiadiazoles nuclei which incorporate toxiphoric –N=C—S– linkage possess anti-inflammatory (Udupi *et al.*, 2000), herbicidal (Nizamuddin *et al.*, 1999), antimicrobial (Demirbas *et al.*, 2005) bactericidal (Sun *et al.*, 1999) and anti-HIV-1 properties(Invidiata *et al.*, 1996).

In this connection, the chemical modification of a natural product isolated from *Cedrus atlantica* essential oil, 1-(4-methylcyclohex-3-enyl) ethanone, using thiosemicarbazide (Paolo *et al.*, 2005; Ourhriss *et al.*, 2005; Aly *et al.*, 2007) followed by treatment of acetic anhydride and pyridine yielded the 1,3,4-thiadiazolic compound (II) with a good yield and high chimiospecifity.

The structure of (II) was established by ^1^H and ^13^CNMR and confirmed by its single-Crystal X-ray structure (Fig. 1).

The thiadiazoline ring may be regarded as having a half-chair conformation with puckering parameters Q= 0.184 (1) Å and φ= 34.1 (4)° (Cremer & Pople, 1975); however it could be also considered as roughly planar with the largest deviation from the mean plane being -0.1069 (8) Å at N1. Such conformation is usual for thiadiazoline rings (Kubota *et al.*, 1982; Radul *et al.*, 2005). The cyclohexenyl ring has a half-chair conformation with puckering parameters Q=0.489 (2) Å, θ= 49.5 (2)° and φ= 344.8 (3)°.

The most interesting feature is the formation of a pseudo ring formed by four molecules associated through N—H···O hydrogen bonds around a fourfold screw axis (Fig. 2, Table 1) so completing a *R*_4_^4^(28) motif (Etter *et al.*, 1990; Bernstein *et al.*, 1995).

### Experimental

To a solution of an equimolecular quantity of compound (I) and thiosemicarbazide dissolved in ethanol, several drops of HCl (*c*) were added. The reactional mixture was heated at reflux during 5 h and then evaporated under reduced pressure. The residue obtained was analysed on silica gel column with hexane: ethyl acetate (95:5) as an eluent. 0.25 mmol of the thiosemicarbazone obtained was dissolved in 2 ml of pyridine and 2 ml of acetic anhydride. The mixture was heated at reflux during 1 h with magnetic stirring, and then evaporated under reduced pressure. The residue obtained was purified on a silicagel column using hexane-ethyl acetate (90:10) as an eluent yielded compound (II) in 60% yield. Suitable crystals were obtained by evaporation of a dichloromethane solution at 277 K. m.p.= 483–484 K (dichloromethane); Spectroscopic analysis: ^1^H NMR (300 MHz, CDCl_3_) δ (p.p.m.): 9.49 (NH, s), 1.80 (3H2, s), 2.07 (1H1', m), 5.57 (1H3', dd, J_1_ = 10 Hz, J_2_ = 6 Hz), 1.58 (3*H*-7', s), 2.13, 2.27 (CH_3_CO, 2 s); ^13^C NMR (75 MHz, CDCl_3_) δ (p.p.m.): 85.4 (C-1), 19.2 (C-2), 36.7 (C-1'), 26.2 (C-2'), 118.1 (C-3'), 132.7 (C-4'), 28.2 (C-5'), 23.0 (C-6'), 22.2 (C-7'), 158.1 (C=N), 169.5, 170.4 (COCH_3_), 22.6, 24.5 (COCH_3_).

### Refinement

All H atoms attached to C and N atoms were fixed geometrically and treated as riding, with C—H = 0.95 (aromatic), 0.98 (methyl) or 0.99 Å(methylene) and N—H = 0.88 Å, with *U*_iso_(H) = 1.2*U*_eq_(C,*N*) or 1.5*U*_eq_(methyl C).

### Crystal data

**Table:**

C_14_H_21_N_3_O_2_S	*Z* = 16
*M**_r_* = 295.40	*F*_000_ = 2528
Tetragonal, *I*4_1_/*a*	*D*_x_ = 1.287 Mg m^−^^3^
Hall symbol: -I 4ad	Mo *K*α radiation λ = 0.71073 Å
*a* = 16.6855 (3) Å	Cell parameters from 9915 reflections
*b* = 16.6855 (3) Å	θ = 2.5–36.1º
*c* = 21.8961 (8) Å	µ = 0.22 mm^−^^1^
α = 90º	*T* = 180 (2) K
β = 90º	Platelet, colourless
γ = 90º	0.29 × 0.24 × 0.08 mm
*V* = 6096.0 (3) Å^3^	

### Data collection

**Table:**

Bruker APEXII CCD area-detector diffractometer	3849 reflections with *I* > 2σ(*I*)
Radiation source: fine-focus sealed tube	*R*_int_ = 0.032
Monochromator: graphite	θ_max_ = 30.5º
*T* = 180(2) K	θ_min_ = 2.4º
φ and ω scans	*h* = −23→23
Absorption correction: none	*k* = −23→23
87517 measured reflections	*l* = −31→31
4637 independent reflections	

### Refinement

**Table:**

Refinement on *F*^2^	Secondary atom site location: difference Fourier map
Least-squares matrix: full	Hydrogen site location: inferred from neighbouring sites
*R*[*F*^2^ > 2σ(*F*^2^)] = 0.037	H-atom parameters constrained
*wR*(*F*^2^) = 0.113	*w* = 1/[σ^2^(*F*_o_^2^) + (0.0505*P*)^2^ + 5.869*P*] where *P* = (*F*_o_^2^ + 2*F*_c_^2^)/3
*S* = 1.11	(Δ/σ)_max_ = 0.001
4637 reflections	Δρ_max_ = 0.39 e Å^−^^3^
185 parameters	Δρ_min_ = −0.26 e Å^−^^3^
Primary atom site location: structure-invariant direct methods	Extinction correction: none

### Special details

**Table:**

Geometry. All e.s.d.'s (except the e.s.d. in the dihedral angle between two l.s. planes) are estimated using the full covariance matrix. The cell e.s.d.'s are taken into account individually in the estimation of e.s.d.'s in distances, angles and torsion angles; correlations between e.s.d.'s in cell parameters are only used when they are defined by crystal symmetry. An approximate (isotropic) treatment of cell e.s.d.'s is used for estimating e.s.d.'s involving l.s. planes.
Refinement. Refinement of *F*^2^ against ALL reflections. The weighted *R*-factor *wR* and goodness of fit *S* are based on *F*^2^, conventional *R*-factors *R* are based on *F*, with *F* set to zero for negative *F*^2^. The threshold expression of *F*^2^ > σ(*F*^2^) is used only for calculating *R*-factors(gt) *etc*. and is not relevant to the choice of reflections for refinement. *R*-factors based on *F*^2^ are statistically about twice as large as those based on *F*, and *R*- factors based on ALL data will be even larger.

### Fractional atomic coordinates and isotropic or equivalent isotropic displacement parameters (Å^2^)

**Table:**

	*x*	*y*	*z*	*U*_iso_*/*U*_eq_	
C1	0.65060 (7)	0.64068 (7)	0.33934 (6)	0.0214 (2)	
C2	0.64575 (8)	0.56723 (8)	0.29765 (6)	0.0287 (3)	
H2A	0.6800	0.5755	0.2618	0.043*	
H2B	0.5902	0.5594	0.2844	0.043*	
H2C	0.6639	0.5197	0.3200	0.043*	
C3	0.68632 (7)	0.78535 (7)	0.31834 (6)	0.0211 (2)	
C11	0.79352 (7)	0.62428 (7)	0.37378 (6)	0.0235 (2)	
C12	0.87096 (7)	0.66585 (8)	0.38792 (7)	0.0281 (3)	
H12A	0.9094	0.6270	0.4043	0.042*	
H12B	0.8615	0.7081	0.4182	0.042*	
H12C	0.8926	0.6897	0.3505	0.042*	
C31	0.62901 (8)	0.90880 (8)	0.27886 (6)	0.0270 (2)	
C32	0.64169 (10)	0.99767 (9)	0.27574 (10)	0.0446 (4)	
H32A	0.6634	1.0120	0.2356	0.067*	
H32B	0.6796	1.0140	0.3076	0.067*	
H32C	0.5904	1.0252	0.2820	0.067*	
C1'	0.61004 (7)	0.62529 (7)	0.40168 (6)	0.0230 (2)	
H1'	0.6342	0.5754	0.4190	0.028*	
C2'	0.52001 (8)	0.61027 (9)	0.39510 (6)	0.0298 (3)	
H2E	0.4950	0.6567	0.3743	0.036*	
H2F	0.5114	0.5624	0.3693	0.036*	
C3'	0.47984 (9)	0.59770 (10)	0.45606 (7)	0.0360 (3)	
H3'	0.4307	0.5690	0.4578	0.043*	
C4'	0.51280 (9)	0.62689 (10)	0.50920 (7)	0.0351 (3)	
C5'	0.58895 (11)	0.66893 (12)	0.50955 (7)	0.0433 (4)	
H5A	0.6287	0.6349	0.5309	0.052*	
H5B	0.5825	0.7184	0.5340	0.052*	
C6'	0.62334 (9)	0.69185 (9)	0.44820 (6)	0.0309 (3)	
H6A	0.6815	0.7023	0.4524	0.037*	
H6B	0.5975	0.7417	0.4336	0.037*	
C7'	0.47125 (12)	0.61447 (13)	0.56956 (8)	0.0504 (4)	
H71	0.4231	0.5818	0.5634	0.076*	
H72	0.4561	0.6665	0.5867	0.076*	
H73	0.5076	0.5869	0.5978	0.076*	
S1	0.604309 (17)	0.726031 (18)	0.298610 (14)	0.02284 (9)	
O1	0.78216 (6)	0.55218 (6)	0.38435 (5)	0.0313 (2)	
O2	0.57194 (6)	0.87583 (6)	0.25536 (5)	0.0338 (2)	
N1	0.73445 (6)	0.66905 (6)	0.34821 (5)	0.0224 (2)	
N2	0.74763 (6)	0.75163 (6)	0.34255 (5)	0.0231 (2)	
N3	0.68691 (6)	0.86761 (6)	0.31029 (5)	0.0251 (2)	
H3	0.7268	0.8951	0.3263	0.030*	

### Atomic displacement parameters (Å^2^)

**Table:**

	*U*^11^	*U*^22^	*U*^33^	*U*^12^	*U*^13^	*U*^23^
C1	0.0182 (5)	0.0197 (5)	0.0262 (5)	−0.0009 (4)	−0.0014 (4)	−0.0010 (4)
C2	0.0296 (6)	0.0251 (6)	0.0312 (6)	−0.0016 (5)	−0.0005 (5)	−0.0063 (5)
C3	0.0177 (5)	0.0211 (5)	0.0245 (5)	−0.0012 (4)	0.0000 (4)	0.0011 (4)
C11	0.0200 (5)	0.0228 (5)	0.0277 (6)	0.0034 (4)	−0.0003 (4)	0.0000 (4)
C12	0.0202 (5)	0.0264 (6)	0.0376 (7)	0.0022 (4)	−0.0047 (5)	0.0019 (5)
C31	0.0242 (6)	0.0267 (6)	0.0302 (6)	0.0032 (4)	0.0007 (5)	0.0060 (5)
C32	0.0397 (8)	0.0253 (7)	0.0687 (12)	0.0039 (6)	−0.0089 (8)	0.0114 (7)
C1'	0.0211 (5)	0.0236 (5)	0.0244 (5)	−0.0011 (4)	−0.0006 (4)	0.0008 (4)
C2'	0.0239 (6)	0.0370 (7)	0.0287 (6)	−0.0066 (5)	−0.0005 (5)	0.0027 (5)
C3'	0.0288 (7)	0.0432 (8)	0.0359 (7)	−0.0025 (6)	0.0059 (5)	0.0077 (6)
C4'	0.0340 (7)	0.0396 (8)	0.0318 (7)	0.0040 (6)	0.0066 (6)	0.0060 (6)
C5'	0.0507 (9)	0.0514 (9)	0.0278 (7)	−0.0088 (7)	0.0052 (6)	−0.0059 (6)
C6'	0.0320 (7)	0.0322 (7)	0.0286 (6)	−0.0054 (5)	0.0009 (5)	−0.0046 (5)
C7'	0.0547 (11)	0.0607 (11)	0.0360 (8)	−0.0001 (9)	0.0146 (8)	0.0057 (8)
S1	0.01844 (14)	0.02350 (15)	0.02657 (15)	−0.00176 (10)	−0.00422 (10)	0.00242 (10)
O1	0.0263 (4)	0.0217 (4)	0.0460 (6)	0.0025 (3)	−0.0046 (4)	0.0038 (4)
O2	0.0284 (5)	0.0368 (5)	0.0361 (5)	0.0003 (4)	−0.0088 (4)	0.0073 (4)
N1	0.0171 (4)	0.0185 (4)	0.0316 (5)	0.0000 (3)	−0.0017 (4)	0.0015 (4)
N2	0.0183 (4)	0.0195 (4)	0.0314 (5)	−0.0011 (3)	−0.0014 (4)	0.0024 (4)
N3	0.0200 (5)	0.0208 (5)	0.0345 (6)	−0.0007 (4)	−0.0038 (4)	0.0035 (4)

### Geometric parameters (Å, °)

**Table:**

C1—N1	1.4897 (15)	C32—H32C	0.9800
C1—C2	1.5303 (17)	C1'—C6'	1.5232 (18)
C1—C1'	1.5451 (17)	C1'—C2'	1.5298 (17)
C1—S1	1.8493 (12)	C1'—H1'	1.0000
C2—H2A	0.9800	C2'—C3'	1.508 (2)
C2—H2B	0.9800	C2'—H2E	0.9900
C2—H2C	0.9800	C2'—H2F	0.9900
C3—N2	1.2822 (15)	C3'—C4'	1.376 (2)
C3—N3	1.3839 (15)	C3'—H3'	0.9500
C3—S1	1.7431 (12)	C4'—C5'	1.451 (2)
C11—O1	1.2396 (15)	C4'—C7'	1.507 (2)
C11—N1	1.3577 (15)	C5'—C6'	1.510 (2)
C11—C12	1.4987 (18)	C5'—H5A	0.9900
C12—H12A	0.9800	C5'—H5B	0.9900
C12—H12B	0.9800	C6'—H6A	0.9900
C12—H12C	0.9800	C6'—H6B	0.9900
C31—O2	1.2141 (17)	C7'—H71	0.9800
C31—N3	1.3710 (16)	C7'—H72	0.9800
C31—C32	1.499 (2)	C7'—H73	0.9800
C32—H32A	0.9800	N1—N2	1.4009 (14)
C32—H32B	0.9800	N3—H3	0.8800
			
N1—C1—C2	112.45 (10)	C3'—C2'—C1'	112.07 (12)
N1—C1—C1'	110.42 (10)	C3'—C2'—H2E	109.2
C2—C1—C1'	111.76 (10)	C1'—C2'—H2E	109.2
N1—C1—S1	102.15 (7)	C3'—C2'—H2F	109.2
C2—C1—S1	107.88 (9)	C1'—C2'—H2F	109.2
C1'—C1—S1	111.79 (8)	H2E—C2'—H2F	107.9
C1—C2—H2A	109.5	C4'—C3'—C2'	121.44 (13)
C1—C2—H2B	109.5	C4'—C3'—H3'	119.3
H2A—C2—H2B	109.5	C2'—C3'—H3'	119.3
C1—C2—H2C	109.5	C3'—C4'—C5'	121.69 (14)
H2A—C2—H2C	109.5	C3'—C4'—C7'	120.61 (15)
H2B—C2—H2C	109.5	C5'—C4'—C7'	117.69 (15)
N2—C3—N3	118.82 (11)	C4'—C5'—C6'	116.78 (14)
N2—C3—S1	118.67 (9)	C4'—C5'—H5A	108.1
N3—C3—S1	122.49 (9)	C6'—C5'—H5A	108.1
O1—C11—N1	119.99 (11)	C4'—C5'—H5B	108.1
O1—C11—C12	122.86 (11)	C6'—C5'—H5B	108.1
N1—C11—C12	117.15 (11)	H5A—C5'—H5B	107.3
C11—C12—H12A	109.5	C5'—C6'—C1'	110.78 (12)
C11—C12—H12B	109.5	C5'—C6'—H6A	109.5
H12A—C12—H12B	109.5	C1'—C6'—H6A	109.5
C11—C12—H12C	109.5	C5'—C6'—H6B	109.5
H12A—C12—H12C	109.5	C1'—C6'—H6B	109.5
H12B—C12—H12C	109.5	H6A—C6'—H6B	108.1
O2—C31—N3	122.57 (12)	C4'—C7'—H71	109.5
O2—C31—C32	122.65 (13)	C4'—C7'—H72	109.5
N3—C31—C32	114.79 (12)	H71—C7'—H72	109.5
C31—C32—H32A	109.5	C4'—C7'—H73	109.5
C31—C32—H32B	109.5	H71—C7'—H73	109.5
H32A—C32—H32B	109.5	H72—C7'—H73	109.5
C31—C32—H32C	109.5	C3—S1—C1	89.42 (5)
H32A—C32—H32C	109.5	C11—N1—N2	117.63 (10)
H32B—C32—H32C	109.5	C11—N1—C1	124.11 (10)
C6'—C1'—C2'	109.00 (11)	N2—N1—C1	116.65 (9)
C6'—C1'—C1	113.93 (10)	C3—N2—N1	110.05 (10)
C2'—C1'—C1	111.97 (10)	C31—N3—C3	123.79 (11)
C6'—C1'—H1'	107.2	C31—N3—H3	118.1
C2'—C1'—H1'	107.2	C3—N3—H3	118.1
C1—C1'—H1'	107.2		

### Hydrogen-bond geometry (Å, °)

**Table:**

*D*—H···*A*	*D*—H	H···*A*	*D*···*A*	*D*—H···*A*
N3—H3···O1^i^	0.88	1.95	2.8223 (14)	171

Symmetry codes: (i) *y*+1/4, −*x*+7/4, −*z*+3/4.
